# Sequencing and Transcriptional Analysis of the Biosynthesis Gene Cluster of Abscisic Acid-Producing *Botrytis cinerea*

**DOI:** 10.3390/ijms151017396

**Published:** 2014-09-29

**Authors:** Tao Gong, Dan Shu, Jie Yang, Zhong-Tao Ding, Hong Tan

**Affiliations:** 1 Key Laboratory of Environmental and Applied Microbiology, Chengdu Institute of Biology, Chinese Academy of Sciences, Chengdu 610041, China; E-Mails: gongtao2007@163.com (T.G.); whosecats@163.com (D.S.); abath@cib.ac.cn (J.Y.); dzhongtaochina@163.com (Z.-T.D.); 2 Henan Key Laboratory of Microbial Engineering, Institute of Biology, Henan Academy of Sciences, Zhengzhou 450008, China

**Keywords:** *Botrytis cinerea*, abscisic acid, comparative sequence analysis, real-time PCR

## Abstract

*Botrytis cinerea* is a model species with great importance as a pathogen of plants and has become used for biotechnological production of ABA. The ABA cluster of *B. cinerea* is composed of an open reading frame without significant similarities (*bcaba3*), followed by the genes (*bcaba1* and *bcaba2*) encoding P450 monooxygenases and a gene probably coding for a short-chain dehydrogenase/reductase (*bcaba4*). In *B. cinerea* ATCC58025, targeted inactivation of the genes in the cluster suggested at least three genes responsible for the hydroxylation at carbon atom C-1' and C-4' or oxidation at C-4' of ABA. Our group has identified an ABA-overproducing strain, *B. cinerea* TB-3-H8. To differentiate TB-3-H8 from other *B. cinerea* strains with the functional ABA cluster, the DNA sequence of the 12.11-kb region containing the cluster of *B. cinerea* TB-3-H8 was determined. Full-length cDNAs were also isolated for *bcaba1*, *bcaba2*, *bcaba3* and *bcaba4* from *B. cinerea* TB-3-H8. Sequence comparison of the four genes and their flanking regions respectively derived from *B. cinerea* TB-3-H8, B05.10 and T4 revealed that major variations were located in intergenic sequences. In *B. cinerea* TB-3-H8, the expression profiles of the four function genes under ABA high-yield conditions were also analyzed by real-time PCR.

## 1. Introduction

Abscisic acid (ABA) is a plant hormone that plays important roles in many aspects of plant growth and development and in the initiation of adaptive responses to various environmental conditions [1,2,3]. It is mainly produced by plants, but also produced by several species of filamentous fungi, such as the genera of *Botrytis*, *Penicillium*, *Cercospora* and *Rhizoctonia*, as a secondary metabolite [4,5]. Strains of *B. cinerea* have been shown to synthesize ABA; overproducing strains are used for biotechnological production of ABA [6,7,8].

Studies on the ABA biosynthetic pathway seem to be different in higher plants and fungi [9]. In higher plants, ABA is derived from the oxidative cleavage of C_40_ carotenoid. Carotenoids, like other isoprenoids, are synthesized from the C_5_ precursor, isopentenyl diphosphate (IPP), which is produced from the 1-deoxy-d-xylulose-5-phosphate (DXP) pathway. IPP is converted to C_20_ geranylgeranyl pyrophosphate (GGPP), from which C_40_ carotenoid was synthesized [10,11]. Most key genes of this pathway, such as genes for DXP synthase, GGPP synthase and carotenoid cleavage dioxygenase, were isolated and intensively studied with ABA-deficient mutants in many species, and the mechanism of this pathway has been well established [12].

In fungi, ^18^O-, ^2^H- and ^13^C-labeling experiments were performed to study the ABA biosynthetic pathway of *B. cinerea* and several *Cercospora* species, and a pathway different from plants has been postulated: IPP, which is synthesized from the mevalonic acid (MVA) pathway, is converted to C_15_ compound farnesyldiphosphate (FPP). Additionally, after a series of reactions of cyclization, isomerization, desaturation and hydroxylation from FPP, ABA is synthesized [13,14]. However different species employ different biosynthetic intermediates. In *C. cruenta*, it has been accepted that ABA was synthesized via 1',4'-dihydroxy-γ-ionylideneacetic acid, while in *B*. *cinerea* and *C. pinidensiflorae*, 1',4'-*trans*-diol-ABA was detected as the main ABA intermediate, and in *C*. *rosicola*, 1'-deoxy-ABA was identified as a more important intermediate than 1',4'-*trans*-diol-ABA [15,16,17,18]. The difference in the biosynthetic pathway suggests that in fungi, the genes for ABA biosynthesis should be quite distinct from higher plants. However, the molecular mechanism driving ABA biosynthesis in fungi is still limited, and only a gene cluster containing four genes (*bcaba1*, *bcaba2*, *bcaba3* and *bcaba4*) in *B. cinerea* was revealed. In the non-sporulating ABA overproducer *B. cinerea* ATCC58025, targeted inactivation of the genes in the cluster suggested at least three genes responsible for the hydroxylation at carbon atom C-1' and C-4' or oxidation at C-4' of ABA, and PCR analysis showed that the organization of *bcaba1-4* is conserved in *B*. *cinerea* ATCC58025 and the highly pathogenic strain B05.10 [19,20]. In contrast to ATCC58025, strain B05.10 does not produce ABA in axenic culture. The mechanisms accounting for the yield diversity of ABA in *B. cinerea* remain enigmatic. Large-scale comparative sequence analysis provides important clues on conserved sequence features, such as genes and regulatory sequences [21]. With the recent progress in the genome analysis of several *B. cinerea* strains, greatly improved opportunities for comparative sequence analysis exist. Most notably, finished or draft sequences of the strains B05.10 [22] and T4 [23,24] genomes are available, giving us a chance to get important clues on conserved sequence features, such as genes and regulatory sequences by comparative sequence analysis [25,26]. Our previous study indicated a hyper-producer *B. cinerea* TB-3-H8 of ABA. The original *B. cinerea* was obtained from wheat stem and leaf and has been improved through a series of mutagenesis and screening over the years. Multiple rounds of mutagenesis and selection generated TB-3-H8 with an ABA productivity of 1.4 g/L [27,28]. The maximum ABA yield of TB-3-H8 increased to 1.8 g/L under optimized conditions. However, the molecular mechanisms behind the strain improvement for higher ABA production are poorly understood. In this study, the ABA biosynthetic gene cluster *bcaba1-4* of TB-3-H8 was sequenced. In addition, the expression levels of the four function genes during ABA production in *B. cinerea* TB-3-H8 have also been investigated. Comparative analysis of the biosynthetic gene clusters of *B. cinerea* strains TB-3-H8, B05.10 and T4 were performed to provide clues for molecular mechanisms of hyper-producing of ABA in TB-3-H8.

## 2. Results

### 2.1. ABA Production of B. cinerea *TB-3-H8*

**Figure 1:**
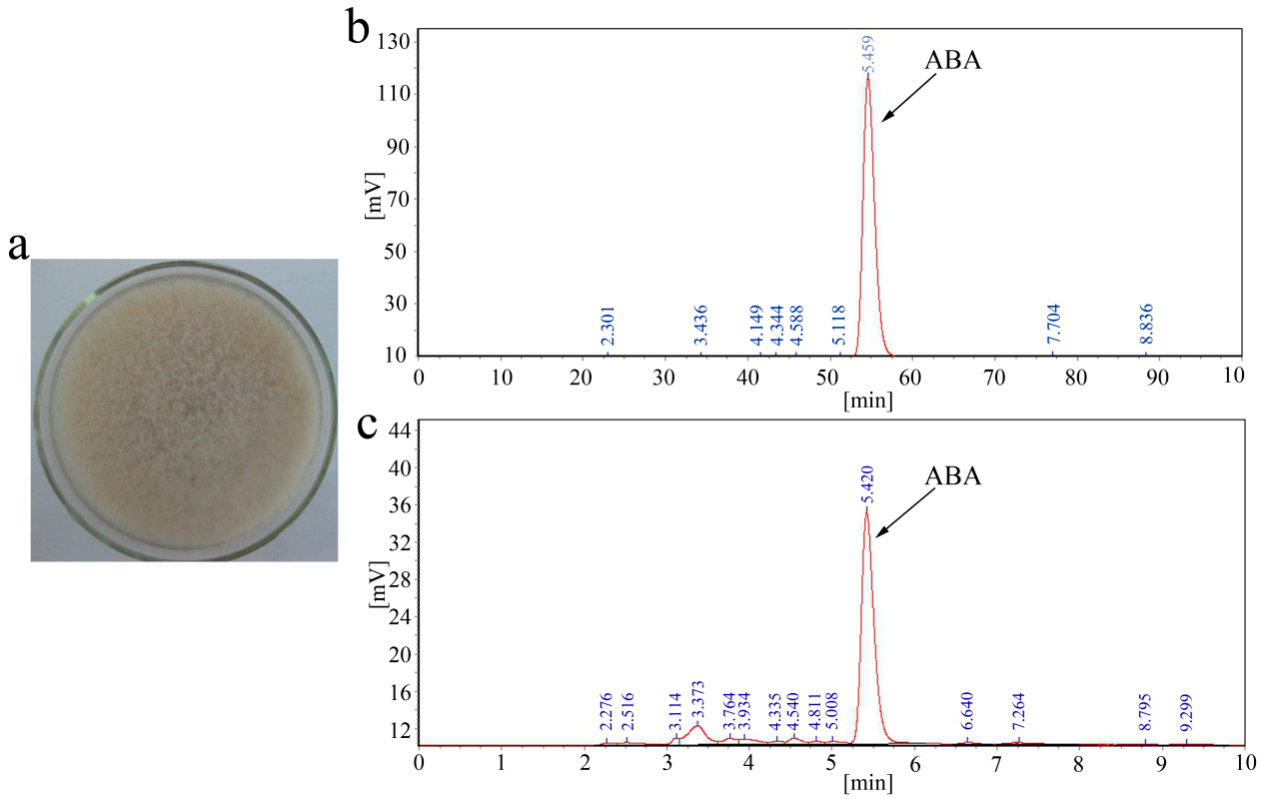
*B. cinerea* strain TB-3-H8. (**a**) Colonies formed by the *B. cinerea* TB-3-H8 on PDA plates 10 days after inoculation; (**b**) HPLC of 98% *w*/*w* ABA; (**c**) HPLC of ABA extracted from the culture medium.

*B. cinerea* TB-3-H8 was a mutant strain obtained after UV treatment of a wild strain originally isolated from wheat stem and leaf. Multiple rounds of mutagenesis and selection generated TB-3-H8 with an ABA productivity of 1.4 g/L [27,28]. The capability of TB-3-H8 to produce ABA was confirmed and quantified by HPLC analysis (Figure 1). Typical time-course profiles of ABA production and cell growth for fed-batch fermentation of TB-3-HB were also studied. Cell growth and ABA production of TB-3-HB in FJ2-FB2 medium with time are presented in later part of the section.

### 2.2. Sequence Analysis of the ABA Cluster of B. cinerea *TB-3-H8*

In view of the large amounts of ABA produced by TB-3-H8, we decided to check for the presence of the ABA gene cluster in the genomes of *B. cinerea* TB-3-H8. The gene cluster was first identified in *B. cinerea* ATCC58025, and targeted inactivation of the genes suggested that at least three genes are responsible for the hydroxylation at carbon atoms C-1' and C-4' or oxidation at C-4' of ABA.

**Figure 2:**
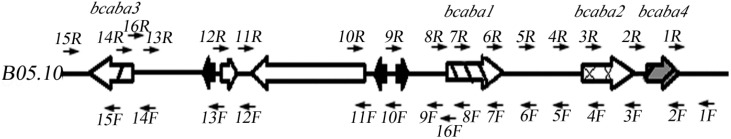
Primer pairs designed according to the sequence in the *B. cinerea* B05.10 genome.

**Figure 3:**
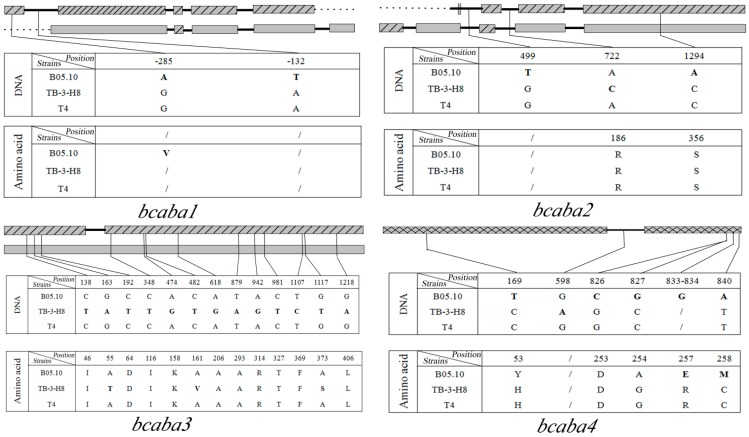
Structure of the ABA gene cluster (*bcaba1*, *bcaba2*, *bcaba3* and *bcaba4*) and 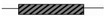 comparison of SNPs among three strains. Sites in TB-3-H8 were used for standard positions of SNPs. Structure of genes in B05.10; 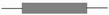 Structure of gene inTB-3-H8 and T4; 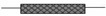 Structure of genes in B05.10, TB-3-H8 and T4, 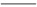 introns, 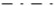 intergenic sequences.

To obtain the sequence of the ABA cluster, we performed several PCRs in strain TB-3-H8 with primers derived from the B05.10 genome (Figure 2). The fragment size of PCR products and sequence analysis revealed the same cluster organization for TB-3-H8 as that of B05.10 and T4 (Figure 3). The sequence homology of the intergenic noncoding regions between cluster genes from TB-3-H8 and B05.10 (or T4) was 73.7% (or 93.3%) on average. Full-length cDNAs were also isolated for the *bcaba1*, *bcaba2*, *bcaba3* and *bcaba4* from TB-3-H8 and compared with the homologous sequences in B05.10 and T4 (Table 1 and Figure 3).

***bcaba3*** is the first gene in the cluster. The full length of this sequence is 1323 bp, containing no introns. It showed that the translated protein had 88% and 99% amino acid identity with the products of the orthologous genes present in the ABA cluster of B05.10 and T4, respectively, and did not show any homology to known sequences as reported in strain ATCC58025. About 600 bps upstream of *bcaba3*, an RNA *pol*III-like promoter was detected.

***bcaba1*:** The full length of this gene is 1769 bps. The five exons were interrupted by four introns, resulting in a protein containing 509 amino acids. The translated protein showed similarities to hypothetical protein P450 monooxygenase [29] and presented 87% and 100% amino acid identity to those of B05.10 and T4, respectively. About 450 bps upstream of *bcaba1*, an RNA polIII-like promoter was also detected.

***bcaba2*:** This 1810-bps gene, which contains four introns, showed similarity to several genes encoding P450 monooxygenases. Analyses of the deduced 527 amino acid sequence performed using the conserved domains database detected that the proteins contain the cypX multi-domains of the P450 superfamily [30]. The deduced protein of *bcaba2* had 83% and 99% amino acid identity with those of B05.10 and T4, respectively. It should be noted that an alternative splicing in the second intron was detected in TB-3-H8 and T4, resulting in an amino acid deletion (Ala) between position 112 and 113 in TB-3-H8.

***bcaba4*:** The last gene of the cluster encodes the short-chain dehydrogenase/reductase. It showed 98% and 100% identity with the deduced amino-acid sequence of *B. cinerea* B05.10 and T4, respectively.

**Figure 4:**
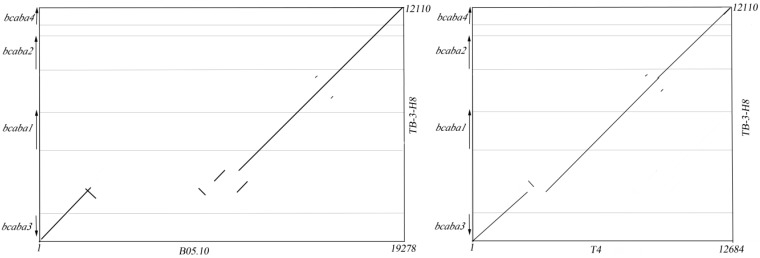
Comparative dot plot analysis of the ABA cluster in *B. cinerea* B05.10 (**A**) and T4 (**B**) with respect to the TB-3-H8 strain.

**Figure 5:**
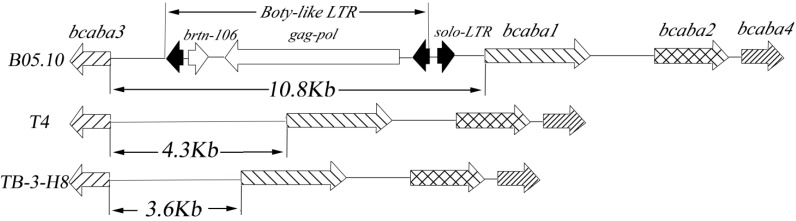
The structure of the ABA gene cluster in *B*. *cinerea* B05.10, T4 and TB-3-H8.

Pairwise dot plot analyses revealed that all four sequences share a high degree of conserved synteny, and some rearrangements were observed (Figure 4). Interestingly, the 12,110-bps TB-3-H8 genomic sequence covered 19,278-bps of the B05.10 sequence. The homologous T4 genomic fragments (12,684-bps) were also smaller in size than the B05.10 sequence. The size difference between TB-3-H8 and the B05.10 genomic fragments is very pronounced as the B05.10 sequences carry a 10,863-bps insertion of two LTR retrotransposon elements between the *bcaba1* gene and the *bcaba3* ortholog (Figure 5).

**Table:** 

Primers	Forward (5'-3')	Reverse (5'-3')	Sequence Length (bp)	
			In B05.10 Supercontig 41	In TB-3-H8 Genome
1	GAGACGGAGAAGCTAAGGAGG	CATGATGGCAATACACCAGTT	1046 (71,207–72,252)	897
2	CCATTTGGTTTGAGCTGCTTTG	CCTCCTTAGCTTCTCCGTCTC	1193 (72,232–73,424)	1193
3	AATTATGCGAAGTCATGTCGA	ACTACCAAAGCAGCTCAAACC	1240 (73,398–74,637)	1240
4	GGGTTTCACTGCGGAGTCTTG	GACTGATCCCATGCGGCAGAT	1221 (74,359–75,579)	1221
5	ACTGTAGCCGAGATAGTAGGG	AGGCTTACGATTGGGCTGGAA	1371 (75,467–76,837)	1386
6	AATGTGGAACTTGCCTTTGGT	CGTTACAACCCTACTATCTCG	933 (76,809–77,741)	933
7	TCGATTCAATCCATATTATGACAAC	AAGTTCCACATTGCGCTCCATCTC	1177 (77,730–78,906)	1177
8	CATAATGTGGGCTAACTACTCTGA	ACCGACCTCCAAGTCAGGCATAT	1034 (78,786–79,819)	1034
9	ATTCGTCTGTGCGTAACCGTGCA	TCAGGTGGTGACGAATACAAGAT	1554 (79,689–81,242)	No data
10	GCAGAAAGGGTGCTCAAAGTGTT	TGCACGGTTACGCACAGACGAAT	986 (81,220–82,205)	No data
11	TTCCTGTGGTCGAGTTTATTG	AACACTTTGAGCACCCTTTCT	4500 (82,183–866,682)	No data
12	TCAGTATGCCTCCTACACGAACA	GCAGCGTACTACAATACGAAACGT	1005 (86,564–87,568)	No data
13	AGGCGGTTCCCTCTTAC	ACGGATACACTCGGCACAACG	2299 (87,402–89,700)	No data
14	ACGATGTTTGTAGAAAGCCACTG	ATCCTCAAGAGTCGCAGTTCATA	1226 (89,625–90,850)	1226
15	CGTCGCTGGGACAACATGCTCAA	AAGGCGAGTGAAAGACGAGATGG	1303 (90,617–91,919)	1303
16	CGCAATGTCAGGAGGGTAGTA	TTATCGCAGAGAATCAGGCAG	10,943 (79,374–90,316)	3762

**Table 1:** *bcaba1-4* genes identified in *B. cinerea*.

Gene Name	Strains Names	GenBank Accession No.	DNA Length (bp)	cDNA Length (bp)	Protein Size (Amino Acids)	No. of Exons	No. of Introns
*bcaba1*	TB-3-H8	/	1769	1530	509	5	4
	B05.10	XM_001553921	1925	1482	493	5	4
	T4	FQ790338.1	1769	1530	509	5	4
*bcaba2*	TB-3-H8	/	1810	1584	527	5	4
	B05.10	XM_001553920	1507	1332	443	4	3
	T4	FQ790338.1	1810	1587	528	5	4
*bcaba3*	TB-3-H8	/	1323	1323	440	1	0
	B05.10	XM_001553924	1323	1269	422	2	1
	T4	FQ790338.1	1323	1323	440	1	0
*bcaba4*	TB-3-H8	/	842	777	258	2	1
	B05.10	XM_001553919	842	777	258	2	1
	T4	FQ790338.1	842	777	258	2	1

The sequence of this band revealed the presence of a solo LTR flanked by 5-bp “CTCAT” target site duplications (TSDs) and a gene encoding a Boty-like retrotransposons (Boty-like LTR) that cannot be found entirely in the corresponding region in *B. cinerea* TB-3-H8 and T4. The Boty-like LTR elements contained two identical LTRs and an internal *gag*–*pol* gene with *brtn-106* [31]. The gene *gag*-*pol* encodes a protein with gag, ZnF_C2HC, RP, RT, IN and CHROMO domains that is potentially functional. The sequence of solo LTR is the same as that of the 5' LTR of the Boty-like LTR, but orientated in the opposite direction (Figure 5).

A deeper look into the genomic information and the surrounding sequences was also undertaken for the genomes of TB-3-H8. Upstream of *bcaba3*, another putative open reading frame shows similarity to a ferulic acid esterase-encoding gene of *Neurospora crassa* as *bcorf2*. The right border of the sequenced DNA region showed similarity to fungal pectin lyase A precursor gene as *bcpl1*. It can be concluded that the organization of the genes from *bcorf2* to *bcpl1* is conserved in *B. cinerea* strains TB-3-H8, B05.10 and T4. This is consistent with the result of Siewers *et al.* mentioned in 2006 [19].

### 2.3. Transcriptional Analysis of the bcaba Cluster

**Figure 6:**
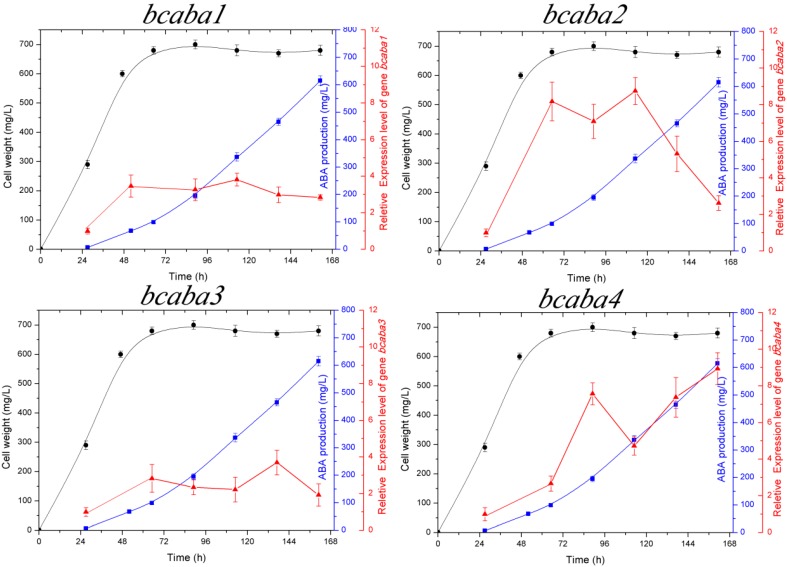
Expression profiles of *bcaba1*, *bcaba2*, *bcaba3* and *bcaba4* in *B. cinerea* TB-3-H8. 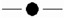 cell weight, 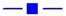 ABA production, 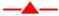 relative expression level of *bcaba1*, *bcaba2*, *bcaba3* and *bcaba4*, respectively.

Real-time PCR was used to study the expression profiles of four genes. RNA isolated from *B. cinerea* TB-3-H8 cells grown in FJ2-FB2 medium was used for real-time PCR analysis. 18S rRNA was used as the internal control for this assay. The expression level was quantified according to the 2^−ΔΔ*C*t^ method. The expression level at 27 h of fermentation was used as a reference and was quantified as 1.0. As displayed in Figure 6, all four genes showed the lowest expression at 27 h, belonging to exponential phase of growth, while each gene expression level increased when ABA began to be produced. The transcript level of *bcaba1* and *2* reached the first peak at 52 h, then decreased and peaked again at 113 h. The transcript level of *bcaba3* increased at 52 h, then decreased until 113 h and peaked again at 137 h. The expression of *bcaba4* appeared to rapidly increase at 89 h, then decreased and reached maximum expression at 161 h.

## 3. Discussions

*B. cinerea* are known as a typical necrotroph, which is responsible for the gray mold disease on more than 200 host plants and have been shown to synthesize ABA [32]. For industrial production of ABA, fermentative conditions using the fungus *B. cinerea* were examined. Irradiation with blue light stimulated the accumulation up to 5.5 mg/100 mL of potato-dextrose agar medium [33]. The microbial synthesis of ABA resulting in the accumulation up to 370.0 mg/g was reported for *B. cinerea* [34]. Strain ATCC58025 of *B. cinerea* is a non-sporulating ABA overproducer, and a gene cluster containing four genes (*bcaba1*, *bcaba2*, *bcaba3* and *bcaba4*) in ATCC58025 was revealed. Targeted inactivation of the genes in the cluster suggested at least three genes responsible for the hydroxylation at carbon atom C-1' and C-4' or oxidation at C-4' of ABA, and PCR analysis showed that the organization of *bcaba1-4* is conserved in ATCC58025 and the highly pathogenic strain B05.10, which do not produce ABA in axenic culture [19].

Our previous study indicated a hyper-producer *B. cinerea* TB-3-H8 with an ABA productivity of 1.4 g/L. In this report, we test the time-course profiles of ABA production and cell growth for fed-batch fermentation of TB-3-HB and found that strains B05.10 and T4 do not produce ABA under our condition (data not published) [35]. A detailed analysis of the ABA cluster *bcaba1-4* in strains TB-3-H8, B05.10 and T4 was also conducted. In some respects, all three clusters look very similar. The genetic organization of the sequenced genes *bcaba1-4* is the same in TB-3-H8, B05.10 and T4. Moreover, the surrounding regions of the ABA cluster are very well conserved in all of the analyzed strains. Two common genes (*bcorf2* and *bcpl1*) were located in all of the available *B. cinerea* genomes that comprise the ABA cluster. The *bcorf2* gene was located upstream in all of the analyzed strains. The downstream region of the ABA cluster is also well conserved.

In contrast to these similarities, all three clusters also exhibit significant differences. The coding regions of the ABA cluster in TB-3-H8 were more similar to that of the T4 strain (≥99% amino acid identity), but considerably different from that of B05.10. Only *bcaba3* of TB-3-H8 contains three amino acid substitutions at positions 55, 161, 373 and an amino acid deletion in *bcaba2* compared to T4. In recent studies, it has been shown that even single amino acid substitutions can be crucial for the functionality of the entire enzyme. Malonek *et al.* previously found that the loss of the ability to produce gibberellins resulted from several point mutations in the coding sequence and promoter regions of certain gibberellin biosynthetic genes [36]. Whether the several point mutations in the coding sequence of *bcaba3* are crucial for the functionality of the entire enzyme has to be elucidated. 

Sequence comparison of TB-3-H8 and B05.10 or T4 genes revealed significantly more differences in the intergenic regions than in the coding regions. The distance between the *bcaba1* and the *bcaba3* gene differs among the genomes of the three strains. The intergenic region between the *bcaba1* and the *bcaba3* gene was also more similar to that of the T4 strain, but different from that of B05.10. Blast analysis showed that the 10,863 bps in B05.10 were homologues to two LTR retrotransposon elements, which were not detected in the strain of TB-3-H8. It is reported that B05.10 shows low expression of *bcaba1* and *bcaba2* under the same conditions in which ATCC 58025 produces ABA. We also found that the levels of *bcaba1-4* in B05.10 and T4 are too low to be detected under the same conditions in which TB-3-H8 produces large amounts of ABA (data not published) [37]. This may be explained by the fact that the presence of the LTR element impeded the transcription of the *bcaba1* or *bcaba3* and other genes, and resulted in an inability to produce ABA. Victor *et al.* previously investigated the biosynthesis gene cluster of putrescine-producing *Lactococcus lactis* [38]. They showed that, in some *L. lactis* subsp. *lactis* strains, the biosynthesis cluster are silenced by the occurrence of one or two IS elements. These examples once again demonstrate the flexibility of nature to achieve differences and similarities via various mechanisms and will surely inspire laboratory efforts to investigate the gene products of the *B. cinerea* ABA cluster. Whether the gene products of the *B. cinerea* ABA cluster in TB-3-H8, B05.10 and T4 are still functional has to be elucidated by further investigations.

In this report, we also investigated the expression levels of the four function genes during ABA production in TB-3-H8. Coregulation is a common feature of fungal metabolic pathway genes. However, it has been shown previously that the four putative ABA genes are not strictly coregulated in ATCC58025 [17]. The expression profile of *bcaba1* shows a persistent induction 90 min after the addition of the ABA precursor, mevalonic acid (MVA), to the medium. A persistent induction could also be detected for *bcaba2*, starting, in contrast to *bcaba1*, after 60 min. Expression of *bcaba3* was likewise enhanced at 60 and 90 min after the addition of MVA, but declined again after 120 min, whereas *bcaba4* was constitutively expressed at a low level [19].

In our work, we found that the addition of mevalonic acid cannot lead to an increased production of ABA obviously in *B. cinerea* TB-3-H8. Therefore, we investigated the expression levels of the four function genes during ABA production in TB-3-H8. The transcript levels of *bcaba1*, *2* and *3* are found to be enhanced at 52 h, then they decrease and peak again at 113 and 137 h, respectively. The expression of *bcaba4* appeared to a rapid increase at 89 h, then it decreased and reached maximum expression at 161 h. It was shown that the expression patterns of *bcaba1-4* have a similar trend over the time course.

## 4. Experimental Section

### 4.1. Strains, Plasmids and Culture Conditions

*Escherichia coli* strain JM109 was used for the propagation of plasmids. The pMD18-T vector (Takara, Otsu, Japan) was used for cloning PCR fragments. *E. coli* cells were grown in Luria-Bertani (LB) liquid broth or on LB agar. *B. cinerea* TB-3-H8 was a mutant strain obtained after UV treatment of a wild strain originally isolated from wheat stem, and leaf. *B. cinerea* strains were grown on potato dextrose agar (PDA) [27]. For the seeding culture, *B. cinerea* TB-3-H8 was grown in YP medium (containing 6.0 g/L glucose, 10 g/L yeast extract, 5.0 g/L soluble starch, 1.0 g/L sucrose, 1.0 g/L NH_4_NO_3_ and 1.0 g/L KH_2_PO_4_) for 48 h at 26 °C and 200 rpm. For ABA production, FJ2 fermentation medium (containing 6.0 g/L glucose, 10 g/L yeast extract, 3.0 g/L soluble starch, 1.0 g/L soybean meal, 2.0 g/L sucrose, 0.5 g/L NH_4_NO_3_ and 1.0 g/L KH_2_PO_4_) and FB2 feeding medium (containing 2.0 g/L glucose, 1.0 g/L sucrose, 5.0 g/L yeast extract and 0.5 g/L soybean meal) was employed. In addition, the two *B. cinerea* (B05.10 and T4) genomes available in databases were also included in this study.

### 4.2. Fed-Batch Fermentation and Determination of ABA

*B. cinerea* TB-3-H8 cultivated on PDA slants for seven days at 26 °C were inoculated into 30 mL fermentation seed medium in 250-mL flasks. After incubation for 48 h at 26 °C and 200 rpm, cultures were trans-inoculated into a 15 L fermentation tank with 8 L fermentation medium by 10% inoculation (*v*/*v*) and grown for 10 days at 30 °C and 200 rpm. The fed-batch fermentation was performed by the continuous supply of sterile feeding medium after 60 h. For quantification of ABA, 25 mL fermentation broth were collected. After centrifuging, the supernatants were analyzed at appropriate dilutions by high performance liquid chromatography (HPLC). ABA (98%, *w*/*w*, Lomon Bio Technology Co., Ltd., Sichuan, China) was used to make the standard curve for quantitative determination. 

### 4.3. DNA Manipulation and PCR Amplification

Total DNA from *B. cinerea* TB-3-H8 used in this work was isolated as described previously by Möller [39]. One µL of the DNA was added to the PCR mix containing 5 μL of supplied PCR buffer, 0.2 mM deoxynucleoside triphosphates (dNTPs), 0.2 mM each oligonucleotide primer, 2 U of KOD-Plus NEO (KOYOTO, Osaka, Janpan) and water in a final volume of 50 μL. PCR amplification was performed using a DNA thermal cycler (Mastercycler; Eppendorf) for 30 cycles with the general program (94 °C for 30 s, 30 s at the suitable annealing temperature of the primers used and 72 °C for 1 min per kb of DNA to be amplified). DNA was separated on a 1% agarose gel in TAE buffer (40 mM Tris-acetate-1 mM EDTA; pH 8.0) and visualized after ethidium bromide staining under UV light conditions. PCR products were purified from an agarose gel by the use of a QIAquick gel extraction kit (Tiangen Biotech. Ltd., Beijing, China).

### 4.4. DNA Sequencing

Total DNA from *B. cinerea* TB-3-H8 was used as DNA templates for sequencing by a polymerase chain reaction (PCR) strategy. Sequencing was performed to identify open reading frames (ORFs) and intergenic regions in the gene cluster involved in ABA formation. Specific primer pairs were made based on the genome of *B*. *cinerea* B05.10. The resulting PCR products were cloned to pMD18-T vector (Takara, Japan) and sequenced. All primers used in this study are outlined in Figure 1.

### 4.5. RNA Extraction

Mycelia were collected at different time points, frozen immediately in liquid nitrogen and ground into powder with a pestle and mortar. RNA isolation was carried out with TRIzol™ (Invitrogen, Carlsbad, CA, USA) following the procedures recommended by the manufacturer. Contaminating DNA was removed by digestion with DNase I (Takara, Japan) and verified by PCR analysis with the RNA as the template. The concentration and integrity of the RNA isolations were determined by both spectrophotometry and agarose gel electrophoresis.

### 4.6. Coding Sequence and Intron Identification by Reverse Transcriptase (RT)-PCR

The first strand of cDNA was synthesized from 20 µg DNaseI-treated RNA with the PrimeScript™ RT reagent Kit (Takara, Japan) and used as the template in PCR. The PCR amplification was carried out as described above [28]. The resulting cDNA fragments were sequenced directly. Intervening sequences (introns) were located by comparison of the genomic DNA to cDNA sequences. Sequence analysis and amino acid prediction/translation have been performed using ClustalW program [40]. All primers used in this study are outlined in Table 2.

**Table 2:** Primer pairs used for cDNA cloning.

Genes	Forward Primers (5'-3')	Reverse Primers (5'-3')
*bcababa1*	5'-ATGTCTAATTCTATATTGAAC-3'	5'-CTATTTGTATTCTGTTCCC-3'
*bcababa2*	5'- ATGCTGCTTAGCATTAAAGA-3'	5'-CTATCTAGGAACCTCTTTTA-3'
*bcababa3*	5'- ATGCAGCAAGTTATTACTCA-3'	5'-CTAGGTACTTTCTCCACGAT-3'
*bcababa4*	5'-ATGTCCTCTCAACCATTCAC-3'	5'-CTAACATCTCCATCCGCCAT-3'

### 4.7. Sequence Analysis

The unmasked *B. cinerea* TB-3-H8 genomic sequences of *bcaba1-4* gene clusters were compared with the corresponding B05.10 genomic sequences by using BLAST on the web server. Promoters were detected with Promoter 2.0 Prediction Serve [41]. Dot plot analysis was done with the program PipMaker [42]. Secondary structures of potential gene products were analyzed with the Bioannotator program from Vector NTI software. For detailed comparisons, local and global pairwise alignments were calculated with the program, AlignX, from Vector NTI software.

### 4.8. Analysis of Gene Expression Profiling by SYBR Green Real-Time PCR Assays

Gene expression profile analysis was carried out by real time-PCR [43,44,45,46]. Amplification was carried out in a Bio-Rad Chromo 4 Real-Time PCR Detection System (Bio-Rad, Hercules, CA, USA) with the Tiangen SYBR Green RealMasterMix (Tiangen Biotech. Ltd., Beijing, China). The 18S rRNA gene was used to correct for sample-to-sample variation in the amount of RNA. The relative mRNA amounts were calculated by the 2^−ΔΔ*C*t^ method from the mean of three independent determinations of the threshold cycle, as described [36]. All primers used in this study are outlined in Table 3.

**Table 3:** Sequences of oligonucleotide primers designed for qPCR.

Genes	Forward Primers (5'-3')	Reverse Primers (5'-3')	Product Size (bp)
*bcaba1*	5'-GCCCAAAGCCTACTGATAAA-3'	5'-TGTCGAATGAATGACCCAAG-3'	124
*bcaba2*	5'-CTTATTACTTCCCGTTTACTC-3'	5'-CTTATTACTTCCCGTTTACTC-3'	101
*bcaba3*	5'-CAAGGAACTCAGCAAGCCC-3'	5'-AGTCGATGCCAACAAAAGG-3'	102
*bcaba4*	5'-CTTGGACGAGTGGGAGTT-3'	5'-GCCGTTGTTAGCCATTAC-3'	91
*18S rRNA*	5'-GAAACTCACCAGGTCCAGA-3'	5'-CAAATCACTCCACCAACTAAG-3'	104

## 5. Conclusions

In summary, this work describes the existence of *B. cinerea* strain TB-3-H8 with the capability to produce ABA. The gene cluster of this pathway has been characterized and was shown to possess the same organization with the other strains, B05.10 and T4. The differences between strains, such as several point mutations in the coding sequence, the presence or absence of an inactivated LTR element, or of two inactivated LTR elements, and the gene expression profiles, reflect different steps in the evolution of *B. cinerea* strains and give us clues to the molecular mechanism of ABA production.
